# Complete genome sequencing and annotation of *Rhodomicrobium vannielii* strain DSM166 suggest affiliation to *Rhodomicrobium lacus*

**DOI:** 10.1128/MRA.00690-23

**Published:** 2023-11-01

**Authors:** Frank D. Müller, Dirk Schüler, Alfons Weig

**Affiliations:** 1 Department of Microbiology, University of Bayreuth, Bayreuth, Germany; 2 Genomics and Bioinformatics, University of Bayreuth, Bayreuth, Germany; The University of Arizona, Tucson, Arizona, USA

**Keywords:** Alphaproteobacteria, Rhizobiales, multicellularity, Hyphomicrobium, *Rhodomicrobium*, MreB

## Abstract

*Rhodomicrobium vannielii* is a multicellular and differentiating member of the order Hyphomicrobiales in the class Alphaproteobacteria. Here, we report the complete genome of strain DSM166 obtained by PacBio SMRT sequencing. The results suggest that this strain is closely related to *Rhodomicrobium lacus*.

## ANNOUNCEMENT

**Fig 1:**
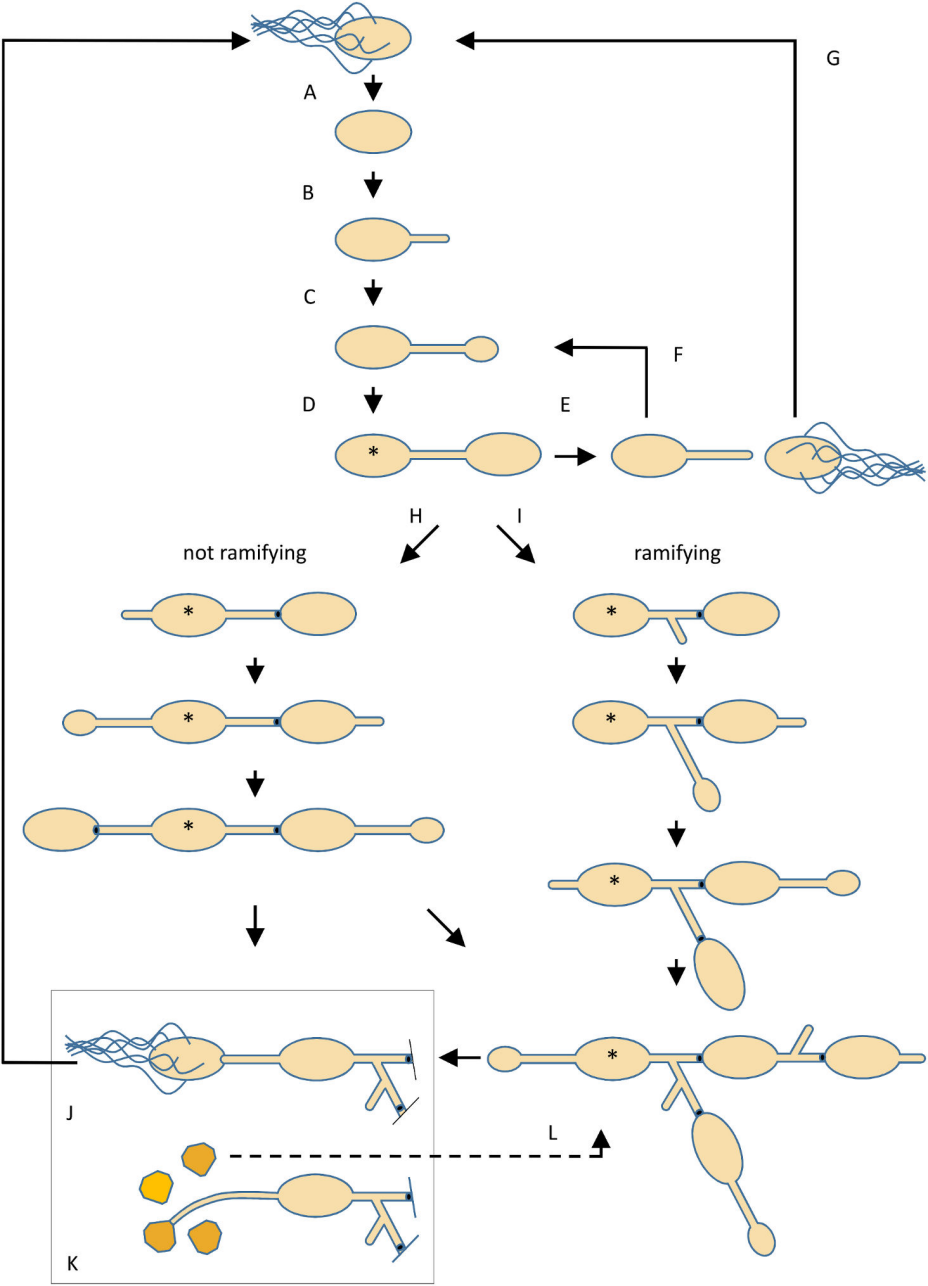
Schematic view of the cell cycles and observed morphotypes described for *Rhodomicrobium vannielii*. (A–G) “Simplified cell cycle” according to Whittenbury and Dow (9) and Dow and France (10). (A) A peritrichously flagellated swimmer cell differentiates most likely irreversibly into a non-motile parental cell. After shedding the flagella and a “maturation period”, a polar hypha is formed (B). Progeny formation starts upon widening of the hyphal tip (C, D). If the offspring cell differentiates into a swimmer and is released (E), the parental cell can initialize the formation of new offspring at the tip of the same hypha (F). The swimmer does not replicate but is destined to settle down at a new place (G) and to enter the cycle as non-motile parental cell (A). (H, I) If the offspring of a parental cell does not differentiate into a swimmer, no fission occurs but the cells remain connected. Further offspring is formed at a hypha that either grows from the distal cell pole or by branching from the most recent hypha, which results in chains (H) or ramified arrays of cells (I). However, it is thought that a parental cell can ever give rise to four offspring cells, and only one offspring cell is formed at a time regardless of how many hyphae are present. Upon maturation of the offspring cell, a “plug” (black dot) is synthesized within the connecting hypha. (J, K) Differentiation of terminal cells from multicellular arrays. Cells can develop either into swarmers (J) or into angular thick-walled exospores (K). Whereas only four spores can be formed by a parental cell, it is not known if the number of swarmers is also restricted. (L) A spore germinates under the outgrowth of one up to four hyphae, and new parental cells are formed at the tip of the hyphae (not shown) leading to multicellular arrays of connected non-motile cells. Stars indicate the oldest cell in an array.

To date, the genus *Rhodomicrobium* consists of three species: *R. vannielii*, *R. lacus*, and *R. udaipurense*. Only the genome of *R. vannielii* ATCC17100 is closed (1–6). Further, yet poorly characterized isolates are deposited in diverse strain collections. The bacteria are described as prosthecate, Gram-negative, photosynthetic, and budding purple nonsulfur bacteria with micro- or anaerobic metabolism found in freshwater habitats. They lack an MreB-based cytoskeleton (7), yet exhibit complex cell morphologies, cell differentiation, and interlaced life cycles comprising single-celled motile and multicellular sessile stages (Fig. 1). Multicellularity and differentiation capabilities suggest complex gene expression and regulation patterns. A genetic system for strain DSM166 has been developed recently (8).

Strain DSM166 from our laboratory stocks was grown at 28°C in 10- or 40-mL W-medium (10 mM HEPES pH 7.0, 15 mM K-lactate, 4 mM NaNO_3_, 0.74 mM KH_2_PO_4_, 0.6 mM MgSO_4_ × 7H_2_O, 3 g/L peptone, and 0.1 g/L yeast extract) in glass bottles on a magnetic stirrer at 1,000 lux light intensity. Cells were harvested from 40 mL by centrifugation and resuspended in 5 mL of 50 mM Tris (pH 8.0) and 50 mM EDTA. Cells were lysed by the addition of lysozyme (10 mg/mL) on ice followed by addition of 1 mL 0.5% (wt/vol) SDS, 50 mM Tris (pH 7,5), 0.4 M EDTA, and 1 mg/mL proteinase K. DNA was isolated using standard phenol-chloroform extraction protocol and ethanol precipitation (11). Precipitated DNA was re-hydrated in 1 mL of 50 mM Tris (pH 7.5) and 1 mM EDTA. Quantity and integrity of DNA were estimated using a Nanodrop spectrophotometer (Thermo Fisher Scientific) and agarose gel electrophoresis.

The extracted DNA was subjected to Novogene’s (UK, www.novogene.com) “Microbial *De novo* Sequencing” service to assemble complex and repetitive genome regions. The Pacific Biosciences (PacBio, CA) Sequel II system was used to generate long reads from circular SMRTbell libraries (PacBio). The reads (consisting of adapters and one to multiple passes around the circular template) were partitioned into adapter-free single-pass reads (206,851 reads; max. read length: 113,597 bp; mean read length: 8,525 bp; and N50: 10,018 bp). These subreads were used for error corrections and genome assembly using Canu (v. 1.9) (12) and Falcon (v. 1.8.1) (13), and the assembly was circularized by Circulator (v. 1.5.5) (14) and rearranged to start at the *dnaA* gene. The long-read-derived assembly was polished using Arrow (v. 2.3.3), and further polished using Pilon (v. 1.23) (15) and Illumina short-reads (4,951,570 paired-end reads; read length: 150 bp); filtered for adapter contamination, >10% uncertain nucleotide or >50% low-quality reads with Q score ≤5). The final genome assembly of 3,739,668 bp was submitted to NCBI’s Prokaryotic Genome Annotation Pipeline (16).

The results of sequencing, assembly, and annotation of the circular DSM166 genome are compared in Table 1 to strains ATCC17100, *R. lacus*, and *R. udaipurense*. Evaluation of the Orthologous Average Nucleotide Identity [OrthoANI (17)] and 16S rRNA genes suggests that *R. vannielii* DSM166 is more closely related to *R. lacus* than to *R. vannielii* ATCC17100 or *R. udaipurense*. A re-classification of this strain may therefore be expected.

**TABLE 1:** Genome characteristics of *R. vannielii* DSM166 compared to other *Rhodomicrobium* spp.

	*R. vannielii* DSM166	*R. vannielii* ATCC 17100, Refseq GCF_016461745.1	*R. lacus*^*a*^, Refseq GCF_003992725.1	*R. udaipurense*^*a*^, Refseq GCF_016461795.1
Genome size (bp)	3,739,668	3,849,085	3,886,079	3,652,920
GC (%)	62.5	62.2	62.4	62.5
Protein	3,268	3,569	3,437	3,317
rRNA	6	6	5	3
tRNA	48	48	45	42
Other RNA	4	4	4	4
Gene	3,382	3,708	3,542	3,479
Pseudogene	56	81	51	113
OrthoANI compared to DSM166 (%)		87.2	97.3	87.1

^*a*^ Genome incomplete/not closed.

## Data Availability

The assembled genome was deposited at NCBI’s genome portal (www.ncbi.nlm.nih.gov) under assembly no. ASM3054524v1, GenBank no. CP125860.1, and RefSeq no. GCA_030545245.1. Raw data are available at NCBI’s SRA portal under BioProject accession no. PRJNA971872 and BioSample accession no. SAMN35056337. Illumina raw reads can be found under SRA accession number SRR24984115 and Nanopore raw reads under SRA accession number SRR24984114.
